# Rotavirus infection among Sudanese children younger than 5 years of age: a cross sectional hospital-based study

**DOI:** 10.11604/pamj.2013.16.88.2519

**Published:** 2013-11-10

**Authors:** Magzoub Abbas Magzoub, Naser Eldin Bilal, Jalal Ali Bilal, Omran Fadl Osman

**Affiliations:** 1 National Public Health Laboratory, Ministry of Health, P.O.BOX 287, Khartoum, Sudan; 2 Department of Medical Microbiology, Faculty of Medical Laboratory Sciences, Khartoum University P.O. Box 11081, Khartoum, Sudan; 3 Pediatric Department, college of Medicine, Qassim University, P.O. Box 6699 Buraydah 51452, Saudi Arabia

**Keywords:** Sudan, rotavirus, children, diarrhoea, acute gastroenteritis

## Abstract

**Introduction:**

In Sudan, rotavirus has been one of the important causative agents of diarrhea among children. Rotavirus A is well known as the leading cause of diarrhea in young children worldwide. It was estimated to account for 41% of hospitalized cases of acute gastroenteritis among children in Sub-Saharan Africa. This study aimed to determine the prevalence and the common clinical presentations of rotavirus A infection among Sudanese children with gastroenteritis seeking management in hospitals.

**Methods:**

755 Sudanese children less than 5 years of age suffering from acute gastroenteritis in hospital settings were included. The positive stool specimens for rotavirus A was used for extract Ribonucleic acid (RNA) and the RNA product was loaded on formaldehyde agarose gel and visualized under UV illumination.

**Results:**

Of the 755 children, 430(57%) were males while 325(43%) were female. The age of children ranged from 1 to 60 months. There were 631 (84%) children who were less than 24 months of age. Out of the 755 stool samples, 121(16%) were positive for rotavirus. Of the 121 infected children with rotavirus, 79(65.3%) were male and 42(34.7%) were female and the highest infection rate was seen among 91(75.2%) of children up to 12 months of age. Children of illiterate parents were more infected with rotavirus than children of educated parents. Severe dehydration present among 70% of infected children with rotavirus.

**Conclusion:**

Since this study is hospital-based, the 16% prevalence rate may not reflect the true prevalence among Sudanese children, thus a community-based surveillance is needed.

## Introduction

Diarrheal diseases are one of the major causes of morbidity and mortality among children less than five years of age. The World Health Organization attributed a worldwide estimate of 17% mortality due to diarrhea in children younger than 5 years of age, 40% of them were in Africa [1]. Rotavirus A is well known as the leading cause of diarrhea in young children worldwide and 82% of rotavirus deaths among children in the poorest countries [2]. Rotavirus estimated to account for 41% of hospitalized cases of acute gastroenteritis among children in Sub-Saharan Africa [3]. The annual rate of rotavirus infection among under-five year old children in the Middle East and North Africa was estimated as 42% [3]. Rotaviruses are members of the Reoviridae family can possess a genome of 11 segments of double stranded Ribonucleic acid (dsRNA) that encode six viral structural proteins and six non-structural proteins [4].

A meticulous research among extensive databases using the MeSh terms; gastroenteritis, rotavirus, children and Sudan yielded no published data except for a local study which was conducted in Melut area (nowadays in the new Republic of South Sudan). Even in this study no prevalence was stated however rotavirus was found to be the second commonest cause of diarrohea among children below five years of age [5].

This study aimed to determine the prevalence and the common clinical presentations of rotavirus A infection among Sudanese children with gastroenteritis seeking management in hospitals.

## Methods

**Nature of the study:** It is a prospective cross sectional hospitals based study conducted in different pediatric hospitals in different states as in and out patients during period from April 2010 to September 2010. The study was including children less than five years of age suffering from acute gastroenteritis (AGE). Hospitals authorities and parents or accompanying care takers of children in the study were informed about the aim and methods of the study.

**Data and samples collection:** A questionnaire was used to collect personal, demographic and clinical data. These included age, gender, residence, symptoms (vomit, diarrhea, fever) and classification of dehydration into either no, mild or severe dehydration. In this study 755 specimens from children less than five years of age with acute watery non-bloody diarrhea were collected. A single stool sample was collected during the attack of acute gastroenteritis (AGE) on the same day and every day up to eight day in a dry, clean, wide mouth screw capped container and placed in an ice chest, then sent directly to the National Public Hearth Laboratory (NPHL), Sudan and preserved at -80 degree centigrade.

**Enzyme Linked Immunosorbent Assay (ELISA):** The samples were brought from -80°C to room temperature (20-25°C). A ratio of 100mg from each stool sample was diluted in 1 ml of samples diluents in a clean dry tube and time shift during pipetting was avoided. VP6 monoclonal antibody (ELISA) was used to detect rotavirus antigen according to the manufacturer's instructions (GA Generic Assays). The Cut-off point was calculated according to the optical density of the mean negative control plus 0.2 as a constant. Samples showing optical densities of more than the set cut-off point were considered positive while those showing optical densities equal or less than the set cut-off point were taken as negative

**Isolation of viral RNA:** The stool samples which were showed positive results by ELISA technique for rotavirus were preserved as raw samples in ice (- 80°C). These samples were used later for RNA extraction. RNA was extracted using the QIAamp^®^ Viral RNA Mini Kit according to the manufacturer's instructions.

**RNA Formaldehyde Agarose (FA) Gel Electrophoresis:** The integrity and size distribution of extracted RNA was checked by denaturing formaldehyde agarose gel electrophoresis and ethidium bromide stain to confirm the ELISA results.

**FA gel electrophoresis of prepared RNA:** Formaldehyde agarose gel electrophoresis was employed for those prepared RNA samples as following: 5?l of 5× RNA loading buffer were added to 20?l of each RNA sample then mixed and incubated for 3-5 min at 65°C then chilled on ice. The chilled samples were loaded onto the equilibrated FA gel wells. The electrophoresis was connected to the electrodes of the electrophoresis apparatus. The RNAs were migrated towards the anode at 5-7 V/cm in 1× FA gel running buffer. The stained gel was illuminated under ultraviolet (UV) light (254-366 nm) allows bands of RNAs to be visualized against a background of unbound dye.

**Statistical Analysis:** The data obtained was coded and entered in to Statistical Package for the Social Sciences software (SPSS). Chi squire test was used to test for significant differences between the variables. A p-value of less than 0.05 was considered as significant.

## Results

### Demographic characteristics of the studied group

The age of overall 755 children their age ranged from 1 to 60 months with a mean of 15.6 ± 13.3 months, both median and mode of 11 months and 631 (84%) children were less than 24 months of age. There were 430 (57%) male while 325 (43%) were females. Table 1.

**Table 1:** Distribution of children according to age and gender

Gender	Male	Female	Total
Age			
0 up to 12 months	263 (61%)	198 (61%)	461 (61%)
More than 12 up to 24 months	95 (22%)	75 (23%)	170 (22%)
More than 24 up to 36 months	34 (08%)	27(08%)	61 (08%)
More than 36 up to 48 months	18 (04%)	15 (05%)	33 (05%)
More than 48 up to 60 months	20 (05%)	10 (03%)	30 (04%)
**Total**	430 (57%)	325 (43%)	755 (100%)

### Prevalence, age and gender characters of rotavirus infected children

Of the 755 stool samples, 121(16%) were positive for rotavirus A antigen of them 79(65.3%) were male and 42(34.7%) were female. This will make a ratio of 1.9:1 (P < 0.05). Table 2. The majority, 85 (70.2%) of rotavirus positive samples were collected after either two or three days of rotavirus infection while 18 samples (14.9%) were collected in the same day or second day of infection and the remaining 18 samples (14.9%) were collected after four or more than four days of rotavirus infection. The represented 70.5% of rotavirus infected children belonged to illiterate mothers whereas 60% belonged to illiterate fathers. The remaining percentages of parents? educational levels were at primary, intermediate, secondary and university levels. There was no statistically significant difference between breastfed and non-breastfed children in relation to rotavirus infection (P > 0.05).

**Table 2:** Distribution of rotavirus infected children according to gender and age

Gender	Male	Female	Total
Age			
0 up to 12 months	59 (75%)	32 (76%)	91 (75%)
More than 12 up to 24 months	08 (10%)	08 (19%)	16 (13%)
More than 24 up to 36 months	08 (10%)	02 (05%)	10 (08%)
More than 36 up to 48 months	02 (2.5%)	00 (00%)	02 (02%)
More than 48 up to 60 months	02(2.5%)	00 (00%)	02 (02%)
**Total**	79 (65%)	42 (35%)	121 (100%)

### Diarrhea, vomiting, fever and dehydration

Of the 755 children, 742(98.3%) had diarrhea, 559 (74.8%) had vomiting and 473 (64%) had fever. Of the 121 rotavirus infected children, 119 (98.3%) had diarrhea, 100(82.6%) had vomiting and 71 (58.7%) had fever. Table 3. 98(81%) of rotavirus infected children had motions of four or five times of diarrhea per 24 hours and 53 (43.8%) with continuous diarrhea for three days, while 91(75.2%) had two, three or four times of vomiting per 24 hours and 56 (46.2%) with continuous vomiting for two days. Eighty six (71.1%) of rotavirus infected children had severe dehydration and 34 (28.1%) had mild dehydration while one child (0.8%) without signs of dehydration

**Table 3:** Distribution of symptoms among rotavirus infected children

Rotavirus infected children	Number	Percent
Children with diarrhea, vomiting and fever	54	44.6%
Children with diarrhea and vomiting	44	36.4%
Children with diarrhea and fever	17	14%
Children with diarrhea only	04	3.3%
Children with vomiting only	02	1.7%
**Total**	121	100%

## Discussion

Detection of rotavirus infection in Sudanese children has been reported in Melut district (nowadays belongs to Republic of South Sudan) but the rate of infection was not stated [5]. In agreement of our prevalence 16% Parashar et al reported 18% of diarrhea in clinical settings due to rotavirus [1]. In contrast to regional and international reports our result is similar to the 17% prevalence reported from Tunisia [6] and Kenya [7] but higher than the 13% prevalence rate reported from Libya [8] and lower than the 21.4% and 32% prevalence rates reported from United Arab Emirate and Brazil, respectively [9, 10]. Moreover the overall 16% prevalence rate fall between the 14 to 45% and 13 to 49% prevalence reported in the Middle East and North African pediatric population [3] and among fifteen African countries around Sudan [11]. Moreover, our figure is higher than the reported from United States and Italy [12, 13]. These differences between our prevalence and the previously mentioned reports may reflect the actual variation in the rates of rotavirus infection. However, it may also be due to different study designs and laboratory tools. Other possible factors such as difference in hygiene and sanitation are important to limit a comparison of rates between those countries. In comparison to the rotavirus infection rate between male and female our males were more willing to get virus than their female in this study which is similar to reported from Italy, Vietnam, Nigeria and Oman [13–15].

Of the infected children with rotavirus, 107(88.4%) were less than 2 years with high prevalence of 88 (72.7%) was found in the age group between 3-12 months. This finding is similar to the same age group rate in Oman [16]. Studies worldwide have reported that the most vulnerable age group to rotavirus infection is under 2 years of age with the highest prevalence between 3- 12 months of age [11, 15, 17, 18]. This is in accordance with the assumption that in under developed areas the early peak of rotavirus may result from early exposure to contaminated sources [20]. The time of samples collection for rotavirus infection is an essential step in molecular characterization of the virus because the viral RNA can easily degrade during delayed collection and delivery of stool samples [19].

The educational background of parents was associated with the rotavirus infected children. The children of illiterate parents showed a high rate of rotavirus infection this may be explained by the fact that the more educated parents will have the skills, practice and knowledge to protect their children from likely exposure to rotavirus.

In agreement to others, in our study no significant difference between breastfed and non-breastfed children in relation to rotavirus infection was detected, a matched case control study tried among Ugandan children concluded that breastfeeding is not protective against rotavirus infection [20]. On the other hand a prospective study in Egypt showed a lower incidence of rotavirus infection in infants fed on breast milk [21]. Another study has evidenced that breastfeeding could offer protection only against severe rotavirus infections [22]. Although this seems to be controversial, it is still possible that breastfeeding may only be protective if it is practiced intensity and frequently. Based on the details of breastfeeding frequency and intensity were not included in this study thus a concrete conclusion on the role of breastfeeding in protection against rotavirus infection cannot be reached.

The findings of Diarrhea, vomiting, fever and their motions of times per 24 hours and lasting for days were similar to findings from Bangladesh, Italy and Tunisia [23–25]. This is in accordance of role of transmission and circulation of virus. The severe and mild dehydration in 120(99.2%) of rotavirus infected children is in accordance with results in Nigerian Italian and Tunisian children population [15, 24, 25].

## Conclusion

The prevalence rate of rotavirus infection among children with gastroenteritis in this study is 16% and the infection rate is higher in males than females. The majority of the infected children were below 2 years of age. This rate is higher in children between 3-12 months and lowest among children less than 3 months of age. Since this study is hospital-based, the prevalence rate will be of value among children with gastroenteritis in hospital populations thus a community-based surveillance will really reflect the true prevalence of rotavirus in Sudan. Rotavirus infection is common among children of illiterate parents. The commonest presenting symptoms were diarrhea, vomiting and fever. Both diarrhea and vominting were encountered in 81% of rotavirus infected children. Severe dehydration is the more common than mild or no dehydration in children with rotavirus gastroenteritis. Breast feeding in this study has no role in protection against rotavirus infection.
